# Isolation and Identification of *Pasteurella multocida* From Bovine Respiratory Disease and the Effect of Baicalin on Its Drug Resistance

**DOI:** 10.1155/vmi/2652895

**Published:** 2026-07-11

**Authors:** Kaiwen Yin, Huiling Zhang, Puguo Hao, Hongliang Fan, Yu Guo, Hongxia Zhao

**Affiliations:** ^1^ College of Veterinary Medicine, Inner Mongolia Agricultural University, Hohhot, Inner Mongolia, 010018, China, imau.edu.cn; ^2^ Ordos Vocational College of Eco-Environment, Ordos, Inner Mongolia, 017010, China; ^3^ Inner Mongolia Yili Industrial Group Co., Ltd., Hohhot, Inner Mongolia, 010010, China; ^4^ Inner Mongolia Autonomous Region Center for Animal Disease Prevention and Control, Hohhot, Inner Mongolia, 010020, China

**Keywords:** baicalin, bovine respiratory disease, drug resistance resensitization, *Pasteurella multocida*

## Abstract

This study aims to clarify the drug resistance status, carriage of drug‐resistant genes, and the effect of baicalin on drug resistance in bovine respiratory *Pasteurella multocida* (Pm). Strains were isolated and cultured from bovine respiratory disease samples, and then PCR identification, drug sensitivity testing, and detection of drug‐resistant genes were carried out. The minimum inhibitory concentration (MIC) of baicalin to Pm was determined by the microdilution method. The effects of baicalin at different concentrations on the drug‐resistant phenotype of Pm were evaluated, and qRT‐PCR was used to measure the impact of baicalin on the expression level of the aac(6′)‐Ib‐cr gene. The fractional inhibitory concentration (FIC) of baicalin combined with enrofloxacin was determined by the microcheckerboard dilution method, and the quinolone resistance–determining region (QRDR) was compared. A total of 10 Pm strains were isolated, including 8 capsular Serotype A and 2 Serotype D; among them, 5 were multidrug‐resistant. All 10 strains carried the strA, strB, and blaROB‐1 drug‐resistant genes. Baicalin at 1/2 MIC and 1/4 MIC concentrations reduced the drug resistance of Pm to amikacin, kanamycin, ciprofloxacin, and enrofloxacin with prolonged intervention time and significantly or extremely significantly decreased the expression level of the aac(6′)‐Ib‐cr drug‐resistant gene. The combination of baicalin and enrofloxacin showed a synergistic effect, and after combined culture, isoleucine (Ile) at Position 83 of the gyrA gene in Pm was replaced by arginine (Arg). The isolated Pm strains were predominantly Serotype A and mostly drug‐resistant. Baicalin can effectively enhance the sensitivity of multidrug‐resistant isolates to amikacin, kanamycin, ciprofloxacin, and enrofloxacin by inhibiting the expression of aac(6′)‐Ib‐cr. The combination of baicalin and enrofloxacin exhibits a synergistic effect, mutually enhancing bacterial sensitivity to the drugs.

Kaiwen Yin, Huiling Zhang, and Puguo Hao contributed equally to this work.

**Novelty Statement**
 
*Pasteurella multocida* (Pm) is a primary bacterial pathogen responsible for respiratory diseases in cattle, posing significant challenges to livestock health and production. The increasing prevalence of multidrug‐resistant (MDR) Pm strains has compromised the efficacy of antibiotic treatments. Baicalin, a natural compound has been extensively reported to exhibit potent antibacterial properties, but limited research has addressed its potential impact on the drug resistance of Pm. This study reveals for the first time at the molecular level the possible mechanism by which baicalin reverses the antibiotic resistance of Pm. Baicalin was found to restore bacterial susceptibility to aminoglycoside and quinolone antibiotics through downregulating the expression of aac(6′)‐Ib‐cr and induction of mutations within the quinolone resistance–determining region (QRDR) of target genes.

## 1. Introduction

Bovine respiratory disease (BRD) is a general term for a large group of diseases caused by bacterial, viral, or mixed infections, as well as many other predisposing factors. It is widely distributed worldwide and is also one of the diseases causing enormous economic losses, with annual losses exceeding 1 billion US dollars [1]. The main pathogen causing BRD, Pm poses a significant challenge to BRD prevention and control. Pm belongs to the *Pasteurella genus* of the *Pasteurellaceae* family and is an aerobic or facultative anaerobic, nonmotile coccobacillus with blunt ends. It is Gram‐negative, and when pathological materials are stained with Wright’s stain or methylene blue, the bacteria exhibit bipolar staining (dark at both ends and light in the middle), existing singly or in pairs. Pm has a capsule but no flagella, and cannot form spores. Currently, the primary serotyping method for Pm is Heddleston’s classification, which is based on differences in capsular antigens [2, 3]. Among them, bovine‐derived Pm is mainly Serotypes A, B, and E. Serotype A Pm can cause fibrinous pneumonia in cattle and is the most prevalent capsular serotype responsible for BRD. Serotypes B and E, on the other hand, lead to hemorrhagic septicemia in cattle [4, 5]. Due to the irrational use of antimicrobial agents in clinical treatment, an increasing number of MDR Pm strains have been detected. With the continuous pressure of antimicrobial agents and the continuous spread of drug resistance, the efficacy of antimicrobial agents in the prevention and control of bovine‐derived Pm has become increasingly poor, which seriously threatens public health safety and poses a great hidden danger to the cattle breeding industry [6–9]. Traditional Chinese medicine (TCM) has unique advantages and is effective for various diseases in animals. The antibacterial active components of TCM are complex, with effective components mainly including alkaloids, flavonoids, terpenoids, and polyphenols. Generally, they can exert antibacterial effects on a variety of bacteria, and a single TCM may contain multiple active components, thus inhibiting multiple bacteria. As the main active component of *Scutellaria baicalensis* (a traditional Chinese herb), baicalin not only embodies the advantages of TCM but also exerts stronger effects [10, 11]. TCM not only demonstrates excellent efficacy against bacterial infection but also reverses drug resistance in established resistant strains to specific antimicrobial agents [12]. The combined use of TCM and antimicrobial agents can reduce the dosage and enhance therapeutic efficacy [13]. Pm is a major bacterial pathogen of BRD, and its antimicrobial resistance has progressively increased under antibiotic selection pressure, posing a significant challenge to clinical control. Baicalin, the principal active compound of *Scutellaria baicalensis*, exhibits well‐established antibacterial, anti‐inflammatory, and antioxidant activities and has been reported to enhance bacterial susceptibility to antimicrobial agents. However, its role in modulating antimicrobial resistance in *P. multocida* remains poorly understood. Therefore, this study aims to collect samples with typical BRD symptoms from large‐scale ranches in northwestern China, determine the drug resistance of isolates, and further investigate the effect of baicalin on the drug resistance of Pm. The research aims to establish a theoretical foundation and provide medication guidance for the clinical treatment of BRD caused by MDR Pm, thereby mitigating negative impacts and economic losses and promoting the healthy and sustainable development of the cattle breeding industry.

## 2. Materials and Methods

### 2.1. Source of Pathological Materials

Pathological materials were obtained from large‐scale ranches in Inner Mongolia, Shanxi, Gansu, and other regions. Lung, liver, and other tissue organs of dead cattle were aseptically collected. Nasal swabs from 54 samples of cattle with respiratory symptoms were also collected.

### 2.2. Main Reagents

Tryptic soy agar (TSA), tryptic soy broth (TSB), nutrient broth medium, and brain heart infusion agar (BHI) were all purchased from Qingdao Haibo Biotechnology Co., Ltd., High‐Tech Industrial Park. Defibrinated sheep blood, newborn calf serum, Ltd. 2 × Taq Plus Master Mix was purchased from Nanjing Novizan Biotech Co., Ltd. Antimicrobial susceptibility test discs were from Hangzhou Microbial Reagent Co., Ltd. Reverse transcription kit and QPCR kit were both from BaoSheng Bioengineering Co., Ltd. Baicalin was obtained from Shanghai Yuanye Biotechnology Co., Ltd.

### 2.3. Main Instruments

Intelligent Biochemical Incubator (SPX‐160) was purchased from Ningbo Jiangnan Instrument Factory. The constant temperature shaking incubator (HZC‐250) was from Taicang Experimental Equipment Factory. PCR (2720 Thermalcycler) was from ThermoFisher. Multifunctional Imager (UVPChemStudio/PLUS) was from Analytik Jena AG, Germany. Real‐time fluorescence quantitative PCR (VIIA7) was from Applied Biosystems, USA.

### 2.4. Isolation and Identification of Pm

Aseptically collected pathological materials were inoculated into 5 mL of nutrient broth medium, incubated at 37°C with shaking at 200 r/min for 16 h in a constant‐temperature shaker, then streaked onto BHI solid medium and blood agar medium. After incubation at 37°C for 12–16 h, bacterial growth and hemolysis were observed. Colonies conforming to typical morphological characteristics of Pm (grayish‐white, smooth, moist, dewdrop‐like, transparent or translucent single colonies with neat edges) were selected for purification culture in TSB containing 5% newborn calf serum. Bacterial DNA was extracted from purified products using the Tiangen Bacterial DNA Extraction Kit as a template for specific gene identification and serological detection of Pm. Primers were synthesized by Sangon Biotech (Shanghai) Co., Ltd., and primer information is shown in Table 1. The reaction system is shown in Table 2. The PCR amplification program was as follows: predenaturation at 95°C for 5 min; denaturation at 95°C for 20 s, annealing at 55°C for 25 s, extension at 72°C for 90 s, 30 cycles; final extension at 72°C for 7 min.

**TABLE 1:** Primer sequence information.

Primer type	Primer name	Primer sequences (5′ ⟶ 3′)	Annealing temperature (°C)	Size of amplified products (bp)	GenBank accession no.
Specific primers	Kmt1	ATCCGCTATTTACCCAGTGG	55	460	AF016259.1
		GCTGTAAACGAACTCGCCAC			

Serotype primer	A	TGCCAAAATCGCAGTCAG	56	1044	CP019081.1
		TTGCCATCATTGTCAGTG			
	B	CATTTATCCAAGCTCCACC	56	760	AF169324.1
		GCCCGAGAGTTTCAATCC			
	D	TTACAAAAGAAAGACTAGGAGCCC	56	657	AF302465.1
		CATCTACCCACTCAACCATATCAG			
	E	TCCGCAGAAAATTATTGACTC	56	511	AF302466.1
		GCTTGCTGCTTGATTTTGTC			
	F	AATCGGAGAACGCAGAAATCAG	55	851	AY604234.1
		TTCCGCCGTCAATTACTCTG			

**TABLE 2:** PCR reaction system (50 μL).

Reagent	Dosage (μL)
2 × Taq MasterMix	25
ddH_2_O	19
F (10 μM)	2
R (10 μM)	2
Template	2

### 2.5. In Vitro Antimicrobial Susceptibility Test

The determination was performed in accordance with the criteria promulgated by the Clinical and Laboratory Standards Institute [14]. The Kirby–Bauer (K‐B) method was used to determine the susceptibility of Pm to 20 antimicrobial agents. The standard strain of *Escherichia coli* ATCC 25922 was used as the quality control bacterium [15]. The criteria for determination are shown in Table 3.

**TABLE 3:** Sensitivity criteria for 20 antibiotics.

Type	Name	Disc content (μg)	Judging criteria (mm)		
			S	I	R
Aminoglycosides	Streptomycin	10	≥ 15	12∼14	≤ 11
	Amikacin	30	≥ 17	15∼16	≤ 14
	Kanamycin	30	≥ 18	14∼17	≤ 13
	Gentamicin	10	≥ 15	11∼14	≤ 12

Quinolones	Ciprofloxacin	5	≥ 21	16∼20	≤ 15
	Ofloxacin	5	≥ 17	14∼16	≤ 13
	Norfloxacin	10	≥ 17	13∼16	≤ 12
	Enrofloxacin	5	≥ 21	17∼20	≤ 16

Tetracyclines	Tetracycline	30	≥ 19	15∼18	≤ 14
	Doxycycline	30	≥ 23	16∼22	≤ 15

β‐Lactams	Amoxicillin	20	≥ 18	14∼17	≤ 13
	Cefotaxime	30	≥ 23	15∼22	≤ 14
	Ceftriaxone	30	≥ 21	14∼20	≤ 13
	Cefepime	30	≥ 15	19∼24	≤ 11
	Sulbactam	10	≥ 15	12∼14	≤ 11

Macrolides	Erythromycin	15	≥ 23	14∼22	≤ 13
	Tilmicosin	15	≥ 14	11∼13	≤ 10

Amphenicols	Florfenicol	30	≥ 19	15∼18	≤ 14

Polypeptides	Polymyxin B	300 IU	≥ 12	9∼11	≤ 8

Sulfonamides	Compound Sulfamethoxazole	23.75/1.25	≥ 16	11∼15	≤ 10

*Note:* S, sensitive; I, intermediary; R, resistant.

### 2.6. Detection of Resistance Genes

Primers for resistance genes were referenced from the literature of Sun et al. and synthesized by Sangon Biotech (Shanghai) Co., Ltd. [15]

### 2.7. Effect of Baicalin on Drug Resistance of Pm

Strains with MDR trends among the isolated bacteria were selected, and the MIC of baicalin against Pm isolates was determined by the microdilution method. Resistance resensitization tests were performed using TSB containing 1/2 MIC, 1/4 MIC, and 1/8 MIC baicalin on Pm. The baicalin was diluted to MIC, 1/2 MIC, and 1/4 MIC in TSB containing 5% newborn calf serum, then mixed with an equal volume of bacterial suspension (1 × 10^8^ CFU/mL). The mixture was cultured at 37°C, and 100 μL of the culture was transferred to fresh TSB medium containing the baicalin concentration every 24 h, accompanied by streak culture, until 72 h. Eliminated strains were obtained after intervention with 1/2 MIC, 1/4 MIC, and 1/8 MIC concentrations of baicalin for 24, 48, and 72 h. The eliminated strains were inoculated into TSB containing 5% newborn calf serum and cultured at 37°C for 12–16 h, and their drug‐resistant phenotypes were determined using the method described in Section 2.5.

### 2.8. Regulation of Baicalin on Resistance Genes of Pm

To determine the effect of different concentrations of baicalin at different intervention times on the resistance gene aac(6′)‐Ib‐cr in the strains selected in Section 2.7, total RNA was extracted using RNAiso Plus (Takara) according to the reagent manual, reverse‐transcribed into cDNA, and subjected to qRT‐PCR detection. The primer information for the reference gene and quinolone resistance gene aac(6′)‐Ib‐cr is shown in Table 4. Using 16S rRNA as the internal reference gene, the relative expression level of the aac(6′)‐Ib‐cr resistance gene in each strain was calculated by the relative quantitative method (2^−ΔΔCt^). Strains before baicalin intervention were used as the control group, and those after intervention as the test group. The calculation formulas were as follows: ΔCt = Ct value of target gene − Ct value of internal reference gene; ΔΔCt = ΔCt (test group) − ΔCt (control group). Data were analyzed and plotted using GraphPad prism 10 and tested by one‐way ANOVA.

**TABLE 4:** Primer information of reference genes and drug resistance–related genes.

Gene name	Sequence (5′ ⟶ 3′)	Annealing temperature (°C)	Size of amplified products (bp)	GenBank accession no.
16S rRNA	AGCGCAACCCTTATCCTTTGT	60	85	PP484884.1
	ATCCCCACCTTCCTCCAGTT			

aac(6′)‐Ib‐cr	GTTTCTTCTTCCCACCATCC	60	103	KM111438.1
	AGTCCGTCACTCCATACATTG			

### 2.9. In Vitro Combined Antimicrobial Susceptibility Test

The MIC of enrofloxacin against Pm was determined by the microdilution method according to the guidelines of the Clinical and Laboratory Standards Institute [14]. The fractional inhibitory concentration (FIC) index of baicalin combined with enrofloxacin was evaluated using the microbroth checkerboard dilution method. Additionally, mutations in the QRDR were detected after cultivation at 1/2 FIC concentration. Primer information is shown in Table 5, and the PCR reaction system is listed in Table 2. The amplification program was as follows: predenaturation at 94°C for 4 min; denaturation at 94°C for 45 s, annealing at 45 s, extension at 72°C for 1 min, 35 cycles; final extension at 72°C for 8 min. PCR products were detected by 1.2% agarose gel electrophoresis, and positive products were sequenced. Sequencing results were aligned using DNAMAN 10 software.

(1) FIC=MIC of TCM Monomers in CombinationMIC of TCM Monomers in Monotherapy+MIC of Antibodies in CombinationMIC of Antibodies in Monotherapy.

**TABLE 5:** Primer information of QRDR genes.

Gene name	Sequence (5′ ⟶ 3′)	Annealing temperature (°C)	Size of amplified products (bp)	GenBank accession no.
gyrA	TAGGGCGTGCATTACCCGATGT	57	407	KP660490.1
	CGCAGGGACTTTAGTTGGGAG			

gyrB	GAAATGACCCGCCGTAA	55	456	KM111337.1
	CTTGCCTTTCTTCACTTTGTA			

parC	TACGAAGGCATTGAACAAAC	55	420	KM111370.1
	CACTGTCCCTTGCCCTAAC			

parE	AATACGGTAAAGCGGTGGC	58	331	KM111403.1
	GGATTCTGCTGGCGGTTC			

The results are determined based on the FIC index as the criterion: A FIC index < 0.5 indicates a synergistic effect, 0.5–1 indicates an additive effect, 1–2 indicates a negligible effect, and > 2 indicates an antagonistic effect.

## 3. Results

### 3.1. Isolation and Identification

A total of 10 Pm strains were initially isolated from 54 BRD pathological samples collected in northwestern China, with an isolation rate of 18.5%. Among them, 8 strains were Serotype A Pm and 2 strains were Serotype D Pm. The strain isolation rates of the pathological samples from each region are shown in Table 6.

**TABLE 6:** Strain isolation rate of pathological samples in each region.

Region	Diseased tissue site	Isolation rate (%)
Inner Mongolia	Lung	18.2% (2/11)
	Nasal swab	9% (1/11)

Gansu	Nasal swab	54.5% (6/11)

Anhui	Nasal swab	4.8% (1/21)

### 3.2. Drug‐Resistant Phenotypes

The drug resistance profiles of 10 bovine respiratory Pm isolates are shown in Table 7. As indicated in the table, 5 Pm strains showed MDR trends, accounting for 50% of the total isolated strains. Specifically, 1 strain was resistant to 4 categories and 8 types of antimicrobial agents, 1 strain was resistant to 4 categories and 6 types, 1 strain was resistant to 3 categories and 7 types, and 2 strains were resistant to 3 categories and 5 types.

**TABLE 7:** Resistance profiles of multiresistant Pm.

Strain name	Number	Type
Pm1	3	S, GM, AMX
Pm2	5	S, CIP, OFX, NOR, AMX
Pm3	7	S, KAN, GM, CIP, ENR, OFX, SXT
Pm4	6	S, AMK, GM, OFX, SXT, AMX
Pm5	4	S, GM, OFX, NOR
Pm6	1	SXT
Pm7	5	S, GM, ENR, OFX, AMX
Pm8	8	S, AMK, GM, CIP, ENR, OFX, SXT, AMX
Pm9	0	—
Pm10	0	—

### 3.3. Carriage of Resistance Genes

The detection results of related resistance genes in 10 Pm strains are shown in Table 8. All isolated strains in this study were universally found to carry the resistance genes strA, strB, and blaROB‐1, with a detection rate of 100%. Some strains carried other resistance genes with the following prevalence rates: qnrD 70% (7/10), aac(6′)‐Ib‐cr 60% (6/10), Sul1 50% (5/10), and tetB 30% (3/10). Less frequently encountered genes included qnrA 20% (2/10), qnrB 10% (1/10), and tetH 10% (1/10). The remaining resistance genes were not detected.

**TABLE 8:** Results of drug resistance gene detection.

Gene	Detection rate (%)	Gene	Detection rate (%)
aadA25	0 (0/10)	tetB	30 (3/10)
aadB	0 (0/10)	tetG	0 (0/10)
strA	100 (10/10)	tetH	10 (1/10)
strB	100 (10/10)	erm42	0 (0/10)
qnrA	20 (2/10)	ermA	0 (0/10)
qnrB	10 (1/10)	ermB	0 (0/10)
qnrC	0 (0/10)	ermC	0 (0/10)
qnrD	70 (7/10)	msrE	0 (0/10)
qnrS	0 (0/10)	mphE	0 (0/10)
oqxA	0 (0/10)	blaTEM	0 (0/10)
oqxB	0 (0/10)	blaOXA‐1	0 (0/10)
aac(6′)‐Ib‐cr	60 (6/10)	blaSHV	0 (0/10)
Sul1	50 (5/10)	blaROB‐1	100 (10/10)
Sul2	0 (0/10)	floR	0 (0/10)
Sul3	0 (0/10)	mcr‐1	0 (0/10)
tetA	0 (0/10)	—	—

### 3.4. Resensitization Effect of Baicalin on Drug Resistance of Pm

#### 3.4.1. Results of Baicalin MIC Determination

The MIC of baicalin was evaluated for six MDR bacterial strains. The value against Pm7 was 1.28 mg/mL, while that against the other five strains (Pm2, Pm3, Pm4, Pm5, and Pm8) was 2.56 mg/mL. All positive controls in the test groups were turbid, while the blank control groups were clear and transparent.

#### 3.4.2. Resensitization Effect of Baicalin on Drug Resistance of Pm

The antimicrobial susceptibility results for Pm treated with different baicalin concentrations for 24, 48, and 72 h are shown in Table 9. As indicated in the tables, baicalin exhibited significantly better elimination effects on aminoglycosides (amikacin and kanamycin) than on quinolones (ciprofloxacin and enrofloxacin). Baicalin showed good effects on resensitizing resistance to amikacin and kanamycin after 24 h of intervention and demonstrated effective elimination of resistance to ciprofloxacin and enrofloxacin after 72 h. The antimicrobial activity of baicalin increased with drug concentration and culture time, indicating a concentration‐dependent effect.

**TABLE 9:** Antibiotic susceptibility results after intervention with different concentrations of baicalin.

Drug name		Amikacin	Kanamycin	Ciprofloxacin	Enrofloxacin
Preintervention sensitivity rate (%)		0 (0/6)	0 (0/6)	0 (0/6)	0 (0/6)

Postintervention sensitivity rate (%) of 1/2 MIC	24 h	83.3 (5/6)	83.3 (5/6)	0 (0/6)	0 (0/6)
	48 h	100 (6/6)	100 (6/6)	33.3 (2/6)	16.7 (1/6)
	72 h	100 (6/6)	100 (6/6)	83.3 (5/6)	83.3 (5/6)

Postintervention sensitivity rate (%) of 1/4 MIC	24 h	83.3 (5/6)	66.7 (4/6)	0 (0/6)	0 (0/6)
	48 h	83.3 (5/6)	100 (6/6)	0 (0/6)	0 (0/6)
	72 h	83.3 (5/6)	100 (6/6)	50 (3/6)	66.7 (4/6)

Postintervention sensitivity rate (%) of 1/8 MIC	24 h	16.7 (1/6)	16.7 (1/6)	0 (0/6)	0 (0/6)
	48 h	33.3 (2/6)	33.3 (2/6)	0 (0/6)	0 (0/6)
	72 h	16.7 (1/6)	33.3 (2/6)	0 (0/6)	16.7 (1/6)

### 3.5. Regulation of Baicalin on Resistance Gene aac(6′)‐Ib‐cr

As shown in Figure 1, after treatment with different concentrations of baicalin, the expression level of the resistance gene aac(6′)‐Ib‐cr generally decreased with the increase in baicalin concentration and culture time. Baicalin at 1/2 MIC concentration significantly downregulated the expression of aac(6′)‐Ib‐cr after 72 h of intervention.

FIGURE 1Effect of baicalin on aac(6′)‐Ib‐cr gene expression in *P. multocida* strains ((A) Pm2, (B) Pm3, (C) Pm4, (D) Pm5, (E) Pm7, and (F) Pm8). Bars represent mean ± SD. ^∗^ *p* < 0.05, *p* < 0.01, and ns = not significant versus control (0 h, no baicalin).

**Figure:**
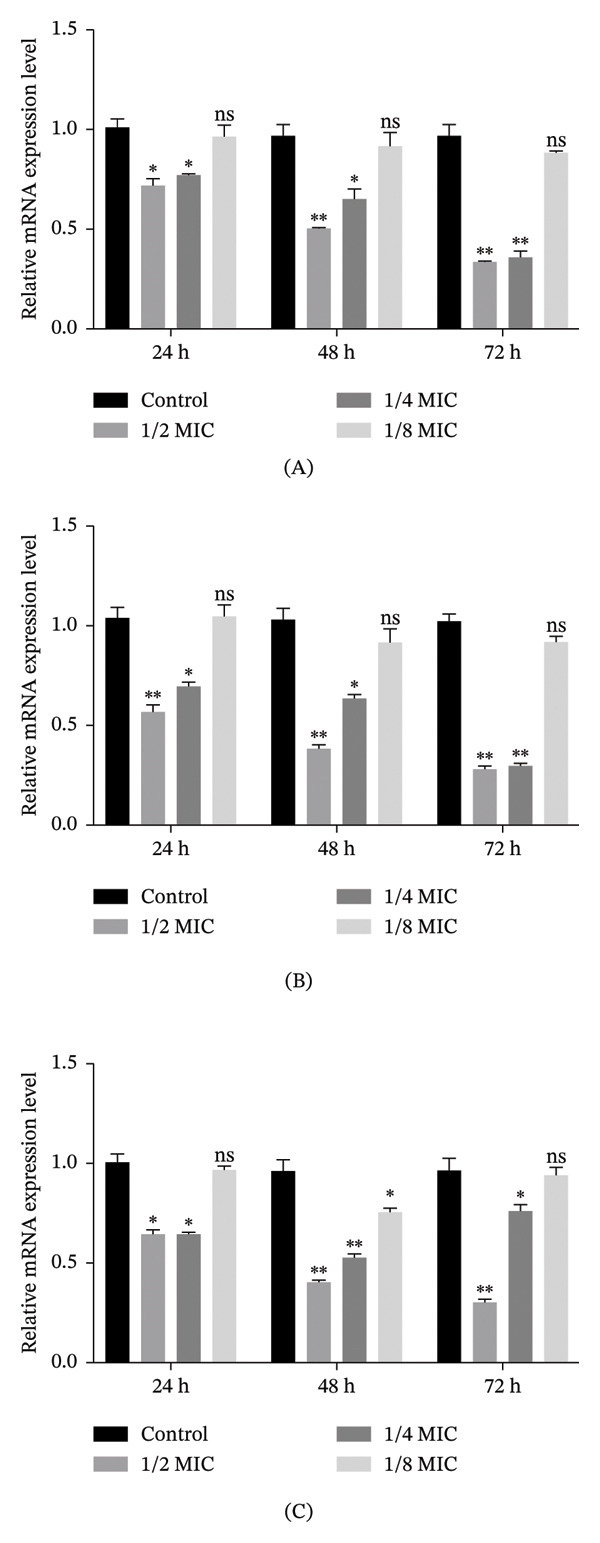

**Figure:**
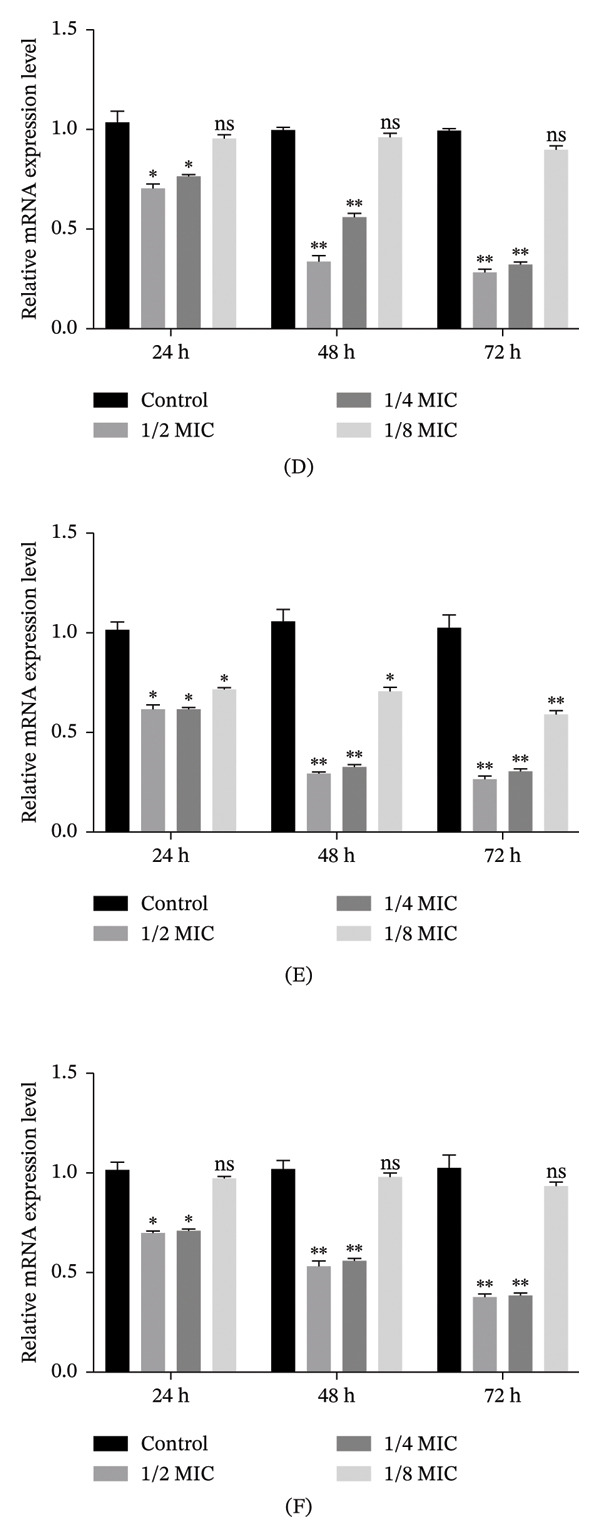

### 3.6. In Vitro Combined Antimicrobial Susceptibility Test

#### 3.6.1. Results of Enrofloxacin MIC Determination

The results of enrofloxacin MIC determination for 6 Pm strains are shown in Table 10. All strains exhibited MIC values of ≥ 0.5 μg/mL, and the maximum MIC (16 μg/mL) was observed for strain Pm8.

**TABLE 10:** The result of MIC of enrofloxacin medicine (unit: μg/mL).

Strain	MIC
Pm2	0.5
Pm3	4
Pm4	0.5
Pm5	0.5
Pm7	8
Pm8	16

#### 3.6.2. Determination of FIC Index for Baicalin Combined With Enrofloxacin

The results are shown in Table 11. When baicalin was combined with enrofloxacin, the FIC indices for Pm2, Pm3, Pm4, Pm5, Pm7, and Pm8 isolates were all less than 0.5. Therefore, the combination of baicalin and enrofloxacin exhibited a synergistic effect against these strains.

**TABLE 11:** Results of the FIC of baicalin combined with enrofloxacin.

Strain	Baicalin MIC (mg/mL)		Enrofloxacin MIC (μg/mL)		FIC	Combined action
	Individual	Combination	Individual	Combination		
Pm2	2.56	0.32	0.5	0.0625	0.25	Synergy
Pm3	2.56	0.16	4	0.25	0.125	Synergy
Pm4	2.56	0.16	0.5	0.125	0.1875	Synergy
Pm5	2.56	0.32	0.5	0.125	0.25	Synergy
Pm7	1.28	0.16	8	1	0.25	Synergy
Pm8	2.56	0.64	16	1	0.3125	Synergy

#### 3.6.3. Effect of Baicalin Combined With Enrofloxacin on QRDR

The results of base mutations and amino acid substitutions in the QRDR gene before and after intervention are shown in Table 12. As indicated in the table, after the combination of baicalin and enrofloxacin, one mutation was found in the gyrA gene of the Pm3 isolate, with corresponding amino acid substitution. There was one base mutation in the gyrA gene (AUA248AGA), and the amino acid was substituted from isoleucine to arginine (Arg) (Ile83Arg).

**TABLE 12:** Results of base mutations and amino acid substitutions in QRDR genes.

Name	Base mutation	Amino acid substitution
gyrA	AUA248AGA	Ile83Arg
gyrB	—	—
parC	—	—
parE	—	—

## 4. Discussion

Pm serves as one of the main pathogens causing BRD. In recent years, the incidence of BRD caused by Pm has been increasing worldwide, imposing substantial economic losses on the cattle breeding industry [16]. In this study, 10 Pm strains were isolated from 54 bovine respiratory pathological samples collected in northwestern China, with an isolation rate of 18.5%. Among them, 8 strains (80%) were Serotype A, and 2 strains (20%) were Serotype D. Bahr and Massieh [17] conducted etiological detection on 246 bovine respiratory samples in Egypt, isolating 32 Pm strains (isolation rate 13%), all of which were Serotype A. Gagea et al. [18] detected pathogens in 54 respiratory disease samples from Canadian cattle farms, showing an isolation rate of 20.4% for Pm, with all capsular serotypes being Type A. These findings are consistent with our results, basically indicating that the prevalence of Serotype A Pm contributes to BRD.

Recent studies have shown that the drug resistance of Pm has gradually increased. Ali et al. tested the drug susceptibility of 77 Pm strains isolated from diseased cattle in Pakistan and found that 54 strains were resistant to trimethoprim, 52 were resistant to erythromycin, and 66 were sensitive to enrofloxacin [19]. Rattanapanadda et al. performed drug susceptibility tests on 381 Pm strains isolated from Canadian cattle, identifying 58 strains resistant to gamithromycin, spectinomycin, tetracycline, florfenicol, and tulathromycin, with a multidrug resistance rate of 21.3% [20]. Credille et al. investigated the drug resistance of pathogens in BRD‐affected cattle in the United States and found that 73% and 63% of 43 Pm strains were resistant to enrofloxacin and florfenicol, respectively, while all were sensitive to ceftiofur and tulathromycin [21]. In addition to its functional role in mediating resistance, the dissemination of resistance genes such as aac(6′)‐Ib‐cr should also be considered. Increasing evidence suggests that antimicrobial resistance genes are frequently associated with mobile genetic elements, including integrative and conjugative elements (ICEs), transposons, and resistance islands, which facilitate horizontal gene transfer among bacterial populations [22]. ICEs, in particular, are capable of integrating into the bacterial chromosome and transferring between cells via conjugation, thereby playing a critical role in the spread of resistance determinants [23]. Although the genomic context of aac(6′)‐Ib‐cr was not investigated in this study, its presence in all resistant isolates suggests a potential association with mobile elements. Therefore, the role of horizontal gene transfer in the dissemination of resistance genes in *P. multocida* warrants further investigation. Furthermore, mechanism by which baicalin downregulates aac(6′)‐Ib‐cr expression is unknown. Baicalin may interfere with stress responses, alter membrane permeability, or directly bind transcriptional regulators. Although flavonoids disrupt membrane integrity and global gene expression [24], direct binding to aac(6′)‐Ib‐cr regulators remains unproven and requires future EMSA and SPR studies.

TCM offers advantages such as wide availability, easy access, low toxic and side effects, and low potential for drug resistance. Zhao et al. found that baicalin effectively resensitizes resistance of *E. coli* to ciprofloxacin and enrofloxacin [12]. This study confirmed that baicalin can effectively reverse Pm resistance to amikacin, kanamycin, ciprofloxacin, and enrofloxacin. Resistance gene detection showed that all six eliminated strains carried the aac(6′)‐Ib‐cr resistance gene. As a mutant of the aminoglycoside resistance gene aac(6′)‐Ib, aac(6′)‐Ib‐cr acquires the ability to encode two different aminoglycoside 6′‐N‐acetyltransferases through two specific amino acid substitutions (Trp102Arg and Asp179Tyr), which acetylate aminoglycosides and quinolones, reducing their activity and leading to bacterial resistance. Machuca et al. showed that carrying aac(6′)‐Ib‐cr increases the MIC of ciprofloxacin by 4–8 fold, significantly reducing its efficacy [25]. The resistance‐reversal effect of baicalin observed in this study may be closely associated with the inhibition of aminoglycoside‐ and quinolone‐modifying enzymes. Our results demonstrated that baicalin intervention significantly or extremely significantly suppressed the expression of the aac(6′)‐Ib‐cr gene. Downregulation of this gene reduces the production of aminoglycoside 6′‐N‐acetyltransferase, thereby diminishing its antibiotic‐inactivating activity and ultimately enhancing bacterial susceptibility to antimicrobial agents. Chan et al. reported that baicalin combined with ciprofloxacin exhibits synergistic effects against ciprofloxacin‐resistant *Staphylococcus aureus*, which is consistent with our results [26]. From a mechanistic perspective, although the present study did not directly investigate the molecular targets of baicalin, increasing evidence suggests that flavonoids, including baicalin, exert antibacterial, and resistance‐modifying effects primarily through indirect modulation of bacterial physiology rather than direct inhibition of transcriptional machinery. Flavonoids have been shown to disrupt bacterial membrane integrity, increase permeability, and interfere with intracellular homeostasis, thereby affecting global gene expression [24, 27]. Moulick and Roy reported that baicalin can inhibit bacterial efflux pump activity, leading to increased intracellular accumulation of antibiotics and enhanced antibacterial efficacy [28]. The inhibition of aac(6′)‐Ib‐cr expression observed in this study may be a secondary effect of baicalin‐induced changes in the intracellular environment, such as increased antibiotic accumulation or cellular stress. However, the exact molecular targets remain to be elucidated, and further studies such as direct binding assay and efflux activity measurement are needed to clarify the mechanism of action.

Mutations in gyrA and parC within the QRDRs are well recognized as major mechanisms of quinolone resistance, primarily by reducing drug binding to the DNA gyrase–DNA or topoisomerase IV–DNA complexes [29].

Kong et al. induced resistance in a bovine Pm isolate using enrofloxacin and ciprofloxacin and analyzed QRDR target mutations, identifying three amino acid substitutions in gyrA (Ser83Ile, Ala84Pro, and Asp87Asn), two in gyrB (Ala418Val and Asp402Asn), and two in parC (Gly78Asp and Glu84Lys) [30]. Among them, Ser83Ile in gyrA and Glu84Lys in parC mediated quinolone resistance in Pm. In contrast to these findings, in our study, the substitution of isoleucine (Ile) to Arg at Position 83 of gyrA in Pm3 restored sensitivity to enrofloxacin, with the MIC decreasing from 4 to 0.25 μg/mL, indicating that this mutation may be associated with altered antimicrobial resistance. Previous studies have demonstrated that high‐level fluoroquinolone resistance typically requires the accumulation of multiple mutations in gyrA, gyrB, parC, or parE, whereas single mutations may confer only limited or variable effects on susceptibility [29]. In the present study, no additional QRDR mutations were detected in parC or other related genes, which may partly explain the relatively low MIC observed in this isolate.

In the present study, the antibacterial and resistance‐reversal activities of baicalin against bovine‐derived Pm were mainly evaluated under in vitro conditions. However, the clinical translation of baicalin in food‐producing animals still faces several challenges, particularly regarding its bioavailability, metabolic stability, and effective delivery to the target tissues. Previous studies have shown that baicalin has relatively low oral bioavailability and can be rapidly metabolized into baicalin and glucuronide conjugates in vivo, which may limit the achievement of effective antibacterial concentrations under practical conditions [31]. Therefore, improving the bioavailability and pulmonary delivery efficiency of baicalin may be important for future applications. Recent studies suggested that nanodelivery systems and inhalation‐based formulations could enhance the stability and tissue distribution of flavonoids in respiratory diseases [32]. In particular, nebulized administration may allow direct delivery of active compounds into the lungs, thereby bypassing gastrointestinal metabolism and improving local therapeutic efficacy [33]. In addition, baicalin, the aglycone metabolite of baicalin, possesses higher lipophilicity and membrane permeability, which may provide superior bioavailability and antibacterial efficacy compared with baicalin itself [34]. Therefore, baicalin may represent a promising candidate for development as a feed additive or inhalable preparation for calves with bovine respiratory disease. This study focused only on aac(6′)‐Ib‐cr expression following baicalin treatment. Although this gene directly associates with the observed aminoglycoside and quinolone resistance, other detected resistance genes (qnrD, Sul1, and tetB) were not investigated for baicalin‐mediated regulation and should be examined in future studies.

## 5. Conclusion

In this study, 10 Pm strains were isolated, showing multidrug resistance primarily against amikacin, kanamycin, ciprofloxacin, and enrofloxacin. The isolates had a high carriage rate of aminoglycoside and quinolone resistance genes, with a certain correlation between drug sensitivity test results and resistance gene carriage. Baicalin at 1/2 MIC and 1/4 MIC significantly reduced the expression of the aac(6′)‐Ib‐cr resistance gene, inhibiting Pm resistance to the above drugs. This restored sensitivity to amikacin and kanamycin in all 6 strains, and to ciprofloxacin and enrofloxacin in 5 strains. The combination of baicalin and enrofloxacin exhibited synergistic effects against Pm, enhancing enrofloxacin sensitivity by inducing the substitution of Ile83 to Arg83 in the gyrA gene.

## Author Contributions

All authors contributed to the study, conception, and design. Funding acquisition, supervision, project administration: Hongxia Zhao. Preliminary disease sample collection and supply: Hongliang Fan and Yu Guo. Data curation: Kaiwen Yin and Huiling Zhang. Formal analysis, Kaiwen Yin and Huiling Zhang. Methodology: Puguo Hao and Hongxia Zhao. Software: Kaiwen Yin, Huiling Zhang, and Puguo Hao. Writing final manuscript and editing: KaiWen, Yin, and Huiling Zhang. Writing–review and editing: Hongxia Zhao.

## Funding

This study was supported by funding from the Inner Mongolia Autonomous Region Science and Technology Major Project (Project No. 2021ZD0013). Inner Mongolia Autonomous Region First Class Discipline Research Special Project (Project No. YLXKZX‐NND‐012).

## Ethics Statement

The current research was conducted according to the rules and regulations of the College of Veterinary Medicine (2022‐2414), Inner Mongolia Agricultural University, Huhhot 010010, Inner Mongolia, China.

## Conflicts of Interest

The authors declare no conflicts of interest.

## Data Availability

The data that support the findings of this study are available from the corresponding author upon reasonable request.
